# Mitochondrial Dysfunction and Sarcopenic Obesity: The Role of Exercise

**DOI:** 10.3390/jcm12175628

**Published:** 2023-08-29

**Authors:** Spyridon Hadjispyrou, Antonios Giannopoulos, Anastassios Philippou, Apostolos Theos

**Affiliations:** 1 Section of Sports Medicine, Department of Community Medicine and Rehabilitation, Umeå University, 901 87 Umeå, Sweden; spyridonas_had@hotmail.gr; 2 Umeå School of Sports Sciences, Umeå University, 901 87 Umeå, Sweden; antonios.giannopoulos@umu.se; 3 Department of Surgical & Perioperative Sciences, Umeå University, 901 87 Umeå, Sweden; 4 Department of Physiology, Medical School, National and Kapodistrian University of Athens, 157 72 Athens, Greece; tfilipou@med.uoa.gr

**Keywords:** sarcopenia, mitochondria, training, physical capacity, VO_2_max

## Abstract

Sarcopenic obesity (SO) constitutes the coexistence of skeletal muscle mass loss (sarcopenia) and excess adiposity (obesity). It is mainly considered as a condition in the elderly with health-threatening impacts ranging from frailty to mortality. Mitochondrial dysfunction consists one of the basic pathophysiological mechanisms leading to the development of SO and its consequences. Indirect indicators of mitochondrial function, such as VO2max and exercise capacity, have been demonstrated to be negatively affected in individuals with SO, while the positive effect of exercise on mitochondrial function has been widely proved; thus, in this review, we aimed at investigating the effects of endurance, resistance, and concurrent exercise training on indexes of mitochondrial dysfunction in SO patients. The results of the clinical trials evaluated reveal positive effects of chronic exercise on VO2max and physical capacity, as well as mitochondrial biogenesis and activity. It has been concluded that utilizing a systematic exercise training program that includes both aerobic and strength exercises can be an effective strategy for managing SO and promoting overall health in these patients.

## 1. Introduction

The term sarcopenia was first coined in 1989 by Irwin Rosenberg and it was inspired by the Greek words “sarx” for flesh and “penia” for loss, referring to the gradual decline in muscle mass that occurs with aging [1,2]. Since then, sarcopenia working groups worldwide have made numerous attempts to establish a unified definition by publishing multiple versions of operational definitions and diagnostics; however, a lack of consensus persists within the scientific community due to the complex nature of its etiology, as well as the unavailability of suitable tools to accurately measure the desired outcomes [3]. The latest revision was made in 2018 by the European Working Group on Sarcopenia in Older people (EWGSOP2) [4]. According to the revised definition and diagnostic criteria, sarcopenia is defined as a progressive and generalized skeletal muscle disorder that is associated with an increased likelihood of adverse outcomes, including falls, fractures, physical disability, and mortality, and is characterized by low muscle strength (dynapenia), low muscle quantity and/or quality, and reduced functional performance [4].

The global prevalence of sarcopenia ranges from 10% to 27% among individuals aged 60 years and older, while the prevalence of severe sarcopenia ranges from 2% to 9% [5]. Men have a higher prevalence of sarcopenia compared to women, and European residents exhibit a higher incidence of developing sarcopenia [5]. As sarcopenia mainly affects the elderly population and the World Health Organization (WHO) predicts that the number of people aged 60 years and older worldwide will almost be doubled, from 1 billion in 2019 to 2.1 billion in 2050, as will also the proportion of elderly compared to those younger than 60 years, it is becoming increasingly important to address the impact of sarcopenia on public health [6].

Although aging and its genetic background are considered to play a regulatory role in the pathogenesis of sarcopenia, this condition is actually multifactorial and results from a complex interplay of various factors beyond aging [7,8,9]. The factors that can contribute to the disturbance of the equilibrium between muscle protein synthesis and degradation include mitochondrial dysfunction, metabolic and endocrine disorders, and neurological factors, as well as age-related physical inactivity and malnutrition [7,9,10]. The complex interaction of these factors can lead to a reduction in the expression of growth factors and elevated oxidative stress and activity of the ubiquitin–proteasome system, as well as increased autophagy and chronic inflammation, ultimately resulting in skeletal muscle impairment and dysfunction [7,11].

Sarcopenia can have a significant impact on the quality of life, and it can be manifested in different ways, ranging from an increased risk of falls and fractures to reduced muscle strength and endurance, cardiorespiratory diseases, and functional decline [4,12,13]. It is a major contributor to frailty and can lead to morbidity, disability, hospitalization, and mortality [4]; however, despite its high prevalence and clinical significance, sarcopenia is often underdiagnosed and undermanaged due to the inhomogeneity and inaccuracy of the screening tools available [4,14,15]. Indeed, sarcopenia was recognized for the first time in 2016 as a distinct disease entity with a specific International Classification of Diseases (ICD-10) diagnosis code (M62.84); however, this implies a promising future regarding its diagnosis and management [16,17].

On the other hand, sarcopenic obesity (SO) has been aptly characterized as the confluence of two epidemics: the aging of the population and the obesity epidemic, which represent two of the most pressing public health concerns in developed countries [18]. It is a condition of the coexistence of abnormal, age-dependent reduced lean body mass (sarcopenia) in the context of excess adiposity (obesity) [10]. While the risk and prevalence of SO tend to increase with age, like sarcopenia, it can occur in individuals of any age [19]. SO affects a significant portion of the older adult population, with an average prevalence ranging from 5% to 10%. The prevalence is even higher in individuals aged ≥80 years. Globally, it is estimated that SO will affect between 100 and 200 million people over the next 35 years, highlighting the need for effective prevention and management strategies [20].

Sarcopenic obesity, as a complex and interrelated syndrome, is characterized by a synergistic effect of sarcopenia and obesity that worsens their health-threatening impacts, eventually leading to an increased risk of mortality [4]. Individuals that have both conditions exhibit a 24% higher risk of all-cause mortality [21], which is the culmination of a cascade of sarcopenia- and obesity-induced effects that include a reduced resting metabolic rate and physical disability, as well as reduced mitochondrial function and oxidative capacity [10]. The pathophysiology of SO involves, among other mechanisms, the dysfunction of mitochondria, which are the energy-producing powerhouse within cells [22]. Mitochondria play a crucial role in maintaining skeletal muscle health and overall metabolic function [23]. In SO, the equilibrium between muscle protein synthesis and degradation is disturbed, leading to muscle wasting, which is often accompanied by mitochondrial dysfunction, reduced mitochondrial mass, impaired energy production, and increased oxidative stress. These mitochondrial abnormalities contribute to the metabolic dysregulation observed in SO [9,10]. Exercise has been shown to improve mitochondrial function in healthy individuals through various mechanisms [24]. This enhancement of mitochondrial function can be observed not only in the healthy population, but also in individuals with SO [25]. In this review, our aim was to investigate the role of exercise on the mitochondrial function in sarcopenic obesity.

## 2. Literature Search Methodology

A systematic, computer-based literature review search with predefined criteria was performed for published clinical trials that addressed the influence of exercise on the mitochondrial function in individuals with SO, using the databases MEDLINE/PubMed of the National Library of Medicine, SCOPUS, and Web of Science. The search methodology used a combination of the following keywords: “sarcopenia”, “obesity”, “sarcopenic obesity”, “mitochondria”, “exercise”, “training”, “VO_2_max”, “physical capacity”, and “basal metabolic rate”. A search of the reference lists of the clinical trials included in this review was also performed for the potential to detect other relevant studies.

The inclusion criteria of this review were papers written in English and peer-reviewed journals concerning the influence of different types of exercise on the mitochondrial function in individuals with SO. Specifically, clinical trials that examined the effects of endurance, resistance, and concurrent exercise training on clinical indicators of mitochondrial function, i.e., on VO2max, BMR, and physical capacity and performance, as well as on mitochondrial biogenesis and activity in individuals with SO were included in this review. 

The exclusion criteria of the review were studies considering the effect of exercise on the mitochondrial function of individuals suffering exclusively either from sarcopenia or from obesity. Clinical trials containing exercise in individuals with SO that examined only molecular indicators of mitochondrial function were also excluded. 

## 3. Sarcopenic Obesity and Mitochondria

The phenotype of SO is not clearly defined and there is no specific clinical presentation; therefore, the disease often goes unrecognized and undiagnosed. Patients usually seek medical care for obesity-related comorbidities, such as type 2 diabetes mellitus (T2DM), non-alcoholic fatty liver disease (NAFLD), dyslipidemia, hypertension, and cardiovascular disease (CVD) [26]. Alternatively, patients may report non-specific symptoms, such as fatigue, weakness, and frailty, which can be attributed to sarcopenia [26]; however, as a general admission, it has been previously demonstrated that individuals suffering from sarcopenia and obesity experience a reduced VO2max and exercise capacity, compared with sex-, race-, and age-matched participants without sarcopenia [27].

The main characteristics of aged skeletal muscle combined with fat accumulation include fiber denervation, expressed as a reduction in the number and size of fast glycolytic type II fibers and recruitment of type I fibers into surviving motor units, due to the loss of motor neurons caused by age-related neurodegeneration [28]. Alterations of the mechanical properties of the muscle–tendon system are subsequently induced [28,29]. The muscle is also affected by fat infiltration, which occurs within or between muscle fibers, referred to as myosteatosis [29,30]. As a consequence of fat accumulation, the production of various adipokines becomes dysregulated, leading to the infiltration of macrophages and other immune cells that generate a wide range of pro-inflammatory cytokines and chemokines, eventually resulting in a chronic low-grade inflammation (“inflammaging”), which has both local and systemic effects [30,31]. The induction of chronic inflammation, in turn, results in further development of sarcopenia, as well as the promotion of insulin resistance, thus inhibiting insulin’s anabolic signals and favoring the development of obesity; thus, a vicious cycle is established between sarcopenia, obesity, and inflammation, leading to the SO phenotype [9,30,31]. 

Notably, it has been reported that SO has a greater negative impact on both metabolic function and physical performance than sarcopenia or obesity alone, making it a critical risk factor for metabolic impairment and physical disability in older age [9,32]. Moreover, it has been demonstrated that SO is a more reliable predictor of physical disability in older individuals rather than either sarcopenia or obesity alone [9,33].

Both aging and obesity share the common feature of impaired skeletal muscle mitochondrial function and chronic inflammation [34]. Several mitochondrial derangements, such as reduced volume, enzymatic activity, respiratory function, membrane dynamics, and quality control, are commonly associated with aging, while the impact of aging on mitochondrial stress is worsened by obesity, which exacerbates the decline in structure and function, as well as protein degradation of mitochondria [35,36]. 

Mitochondrial dysfunction in skeletal muscle has a profound impact on muscle mass regulation, which operates through interconnected pathways, including the mitochondrial proteolytic system, dynamics (fusion/fission), and mitophagy [37,38]. Beyond their role in energy production, mitochondria play a central part in muscle homeostasis by influencing calcium buffering and regulating nuclear gene programs for muscle mass [38]. Alterations in mitochondrial distribution, morphology, and function are present in atrophic muscles in aging, muscle disuse, insulin resistance, and other diseases [39]. Maintaining mitochondrial quality is crucial, as skeletal muscle is a post-mitotic tissue and cannot dilute damaged mitochondria through cellular division. Consequently, the dysregulation of mitochondrial quality control pathways can trigger catabolic signaling, leading to muscle loss. The disruption of mitochondrial fusion and fission significantly affects muscle development, maintenance, and function, resulting in muscle atrophy, weakness, and far-reaching consequences for the overall physiology of the body due to the role of skeletal muscle as an endocrine organ [38]. 

Due to the highly interconnected and dynamic communication between adipose tissue and skeletal muscle tissue, and as obesity is characterized by an excessive accumulation of lipids at the adipose tissue, dysfunctional skeletal muscle in SO exhibits accumulation of these lipids and their derivatives in the form of both inter- and intra-myocellular free fatty acids [9]. This deposition of myocellular lipids induces mitochondrial dysfunction and a reduction in the number of mitochondria, as well as a disturbed β-oxidation of free fatty acids, which in turn promotes the production of reactive oxygen species (ROS) and oxidative stress [10,29,40]. As a result, lipotoxicity, insulin resistance, and the secretion of pro-inflammatory myokines are established, which affect muscle tissue function [7,29]. Myokines, in turn, worsen inflammation in adipose tissue by their endocrine/paracrine effects, creating a harmful, vicious cycle between mitochondrial dysfunction and inflammation in both muscle and adipose tissue, triggering the pathological cascade of SO [9,29,40]; thus, the disruption of muscle protein synthesis, the impairment of skeletal muscle function, and the reduction of muscles’ oxidative capacity cannot been overcome, consequently establishing the condition of SO [9,10].

In laboratory settings, the assessment of mitochondrial function and respiration has been primarily used, measuring the activity levels of key oxidative enzymes involved in oxidative phosphorylation (OXPHOS), such as citrate synthase (CS) and cytochrome C oxidase (COX-IV) of the electron transport chain [41]; however, implementing these laboratory-based techniques for a routine clinical assessment of mitochondrial function in every patient would be non-practical and infeasible. Hence, indirect indicators of mitochondrial function have been utilized by clinicians to measure metabolic flexibility and infer mitochondrial function, such as VO2max, basal metabolic rate (BMR), and physical capacity [42]. This is because greater muscle oxidative capacity has been shown to be related to higher VO2max and higher BMR, as well as higher physical capacity [42,43]. These factors indicate the role of mitochondria in energy production and expenditure, and that the number and function of mitochondria are important determinants of these measures of energy metabolism and physical performance [22]. In investigating these indicators in the SO population, it has been shown that these patients exhibit decreased values for VO2max, BMR, and physical capacity [27,44]. These findings suggest that SO is closely associated with mitochondrial dysfunction, and impaired mitochondrial function in SO patients contributes to decreased physical capacity and an increased risk of metabolic disorders. 

## 4. Exercise as a Treatment

SO is a condition that poses significant challenges for clinicians, as no clear guidelines have been established for its treatment; however, there is a growing research interest in the treatment of SO and promising interventions have emerged in the field, primarily divided into two categories: pharmacotherapy and non-pharmacotherapy [10,45]. The simultaneous pursuit of gaining skeletal muscle mass and losing fat mass is required for achieving optimal results [29]. Some of the pharmacotherapy options consist of testosterone and selective androgen receptor modulation (SARM), which can promote hormonal manipulation and inhibit sarcopenia-associated factors, as well as myostatin monoclonal antibodies, which have been shown to be promising by inhibiting skeletal muscle mass loss and enhancing skeletal muscle growth and physical function [10,29]. Non-pharmacotherapy options, such as nutrition/dietary strategies and exercise/physical activity interventions, remain the hallmark of treatment options. Specifically, nutrition/dietary strategies, such as calorie restriction, vitamin D and calcium supplements, and high-quality protein intake, as well as exercise/physical activity, including aerobic and resistance training or their combination, have shown a significant potential for improving SO [10,45].

Among the aging population, individuals with sarcopenia and obesity typically experience a decrease in physical activity, or aerobic and muscle-strengthening exercise, which raises concerns about the quality of their extended life expectancy. This decline can be attributed to several factors, including possible discomfort and pain associated with exercise, a reduction in occupational activity post-retirement, osteoarthritis and musculoskeletal injuries, fear of falling and increased risk of fractures due to osteoporosis, and decreased interest and physical ability in sports and exercise [46,47]. When considering the benefits of exercise, especially for the elderly and individuals with sarcopenia and obesity, it becomes clear that incorporating exercise into their daily routine is necessary, while adhering to appropriate guidelines and considering any comorbidities [10,29,47]; however, exercise should be considered a distinct subcategory of physical activity, involving planned, structured, and repetitive movements aimed at improving or maintaining physical fitness, whereas physical activity is any bodily movement produced by skeletal muscles that require energy expenditure [48]. Particularly for muscle mass maintenance, personalized and periodized strength-based resistance training is crucial, especially for the elderly population. While low-intensity exercises are commonly prescribed, strength-based training, when progressively performed under supervision, is more effective in increasing muscle strength and hypertrophy [49].

According to WHO, in order to maintain good health and well-being, all adults should engage in a minimum of 150 to 300 min of moderate-intensity aerobic exercise per week, or an equivalent amount of vigorous activity [50,51]. Additionally, for older adults (aged 65 years and older), incorporating two non-consecutive sessions of resistance training that focus on improving balance, coordination, and muscle strength can be beneficial in reducing the risk of falls and promoting overall health [50,51]. Exercise is generally safe for the elderly population, even when they have comorbidities; however, it is important to follow a properly designed, potentially individualized exercise training program that includes adequate instructions and possibly supervision [9,45,46,47]. Apart from the various advantages that exercise provides, including its ability to reduce blood pressure, enhance cardiorespiratory fitness, improve insulin sensitivity, and aid in weight management, the exercise interventions utilized in the treatment of SO aims, among other objectives, at enhancing mitochondrial function [51,52,53,54].

Indeed, it is now well recognized that exercise is a potent stimulus for activating signaling pathways that produce robust phenotypic changes in the mitochondrial milieu, improving mitochondrial function and leading to greater muscle health [55]. This relationship has been recognized since 1973, when Harper et al. revealed the positive correlation between VO2max and mitochondrial volume [56]. As VO2max reflects the maximum amount of oxygen that an individual can consume during exercise, which is required by mitochondria to produce ATP through oxidative phosphorylation, the more mitochondria a muscle has, the more oxygen it can use during exercise, and the higher the VO2max [56]. Physical exercise, whether aerobic, resistance training, or a combination of both, is known to promote mitochondrial dynamics, increasing the total mitochondrial proteins and improving oxidative phosphorylation and respiratory capacity per mitochondrion, including the CS, the COX-IV, and specific substrates of the OXPHOS system, even in the elderly population [55,57]. Exercise interventions also improve mitochondrial antioxidant capacity and promote mitochondrial density, in terms of mRNA and the enzymes involved in mitochondrial biogenesis, such as peroxisome proliferator-activated receptor-gamma coactivator-1 alpha (PGC-1a) [57]. Through these pathways, exercise is proposed to significantly benefit patients with SO by targeting mitochondrial function and, thus, physical performance is suggested to be a potential tool for categorizing the severity of sarcopenia [4]. The ability of exercise to mediate various biological effects on the dysfunctioning mitochondria in SO patients and regulate oxidative stress, mitochondrial biogenesis, and skeletal muscle oxidative functioning makes exercise one of the most promising interventions for treating SO [25,37,47]. Table 1 presents studies that examined the effects of various types of exercise on individuals with SO, focusing on the outcomes related to mitochondrial function and their functional impact in those patients.

Exercise plays a pivotal role in addressing SO by countering the effects of mitochondrial dysfunction and regulating muscle mass, and its effectiveness lies, in part, in its ability to enhance mitochondrial plasticity [37]. Aging often leads to dysregulation in the cellular pathways responsible for mitochondrial turnover, resulting in the accumulation of damaged organelles within muscle cells. This compromised mitochondrial quality activates myonuclear cell death and leads to muscle atrophy [37,38]; however, exercise, both acute and chronic, mitigates these deficits by restoring mitochondrial turnover through various mechanisms. Exercise triggers signaling pathways that upregulate PGC-1α and other transcription factors responsible for mitochondrial biogenesis, increasing mitochondrial content and function. In addition, it influences mitochondrial morphology by promoting the abundance of fusion and fission proteins, facilitating mitochondrial turnover to clear damaged mitochondria and dissipate energy throughout the muscle cell. Furthermore, exercise alters autophagy markers, leading to enhanced autophagosome formation and degradation of damaged mitochondria via mitophagy [71]. Lastly, exercise-induced improvements in mitochondrial function reduce pro-apoptotic factors and inhibit apoptosis-related signaling cascades, ultimately preserving the myofiber number and size during aging [37,38].

### 4.1. Endurance Training and Its Effects on the Mitochondria in Sarcopenic Obesity

Endurance exercise has been widely used to enhance physical fitness and promote overall health [72]. It prevailingly uses aerobic metabolism and thus stimulates mitochondrial biogenesis and improves mitochondrial function and efficiency [73]. Specifically, aerobic activity leads to an enhancement of the oxidative capacity of skeletal muscle by increasing the size and number of mitochondria per unit area and increasing the concentration of the electron transport chain and Krebs cycle enzymes, as well as by counteracting the negative effects of intra-myocellular lipids and accelerating lipolysis, which consequently reduce oxidative stress and enhance mitochondrial respiration [10,72,73]. Moreover, it has been found that endurance exercise training can potentially stimulate muscle protein synthesis through modulation of the mTORC1 pathway, reduce muscle protein breakdown by regulating the ubiquitin proteasome pathway, and enhance mitochondrial biogenesis [74]. Consequently, these effects lead to the promotion of a positive net protein balance and muscle hypertrophy [7,74]. The positive effects of endurance exercise on muscle and mitochondrial quality and function play a crucial role in combating the age-related decline in muscle mass and function associated with SO [10,73]; therefore, incorporating aerobic exercise into the lifestyle of older adults with SO could yield substantial benefits, optimizing mitochondrial function and contributing to the improvement of overall health and physical performance [10].

Indeed, Villareal et al. 2017 conducted a 26-week clinical trial on 120 obese and frail adults to compare the effectiveness of endurance training, resistance training, and concurrent training, along with a weight-management program. The endurance exercise group showed a 14% increase in the physical performance test (PPT) score, which was lower than that of the concurrent training group but equal to that of the resistance exercise training group. Additionally, VO2max showed an 18% increase after the 26-week intervention, which was higher than that of the concurrent and the resistance training groups [58]. Moreover, Colleluori et al. (2019) conducted a clinical trial to compare the effects of different exercise regimens (endurance, resistance, and concurrent) combined with matched calorie restriction on muscle protein synthesis and myocellular quality in 35 elderly individuals with SO over a 6-month intervention period. The endurance group showed a 13% increase in the PPT score, which was lower than that of the concurrent group but equal to the resistance group and higher than that of the control group. Additionally, VO2max showed a 16% increase after the 6-month intervention, which was higher than that of the resistance group and equal to the concurrent group. Furthermore, the endurance group exhibited greater increases in the expression of mitochondrial fusion and fission, as well as chaperone proteins, potentially because of the higher enhancement observed in mitochondrial bioenergetic regulators and mitochondrial activity (indicated by increased CS and COX-IV activities). Finally, the endurance group exhibited the highest increase in the expression of transcription factor A-mitochondrial (TFAm), a factor that participates in mitochondrial biogenesis, compared to the other training groups [59]. In addition, Chen et al. (2017) designed an RCT to investigate the influence of different kinds of exercise (endurance, resistance, and concurrent) on the insulin-like growth factor-1 (IGF-1) levels and muscle performance in 45 elderly adults with SO over an 8-week period. The blood levels of the anabolic factor IGF-1 were superior to the control group, but they were significantly lower in the endurance training group compared to the concurrent training group. Moreover, the endurance training group, along with all the other training groups, exhibited superior muscle strength compared to the control group [60]. 

### 4.2. Resistance Training and Its Effects on Mitochondria in Sarcopenic Obesity

The utilization of resistance exercise has proven to be an efficient intervention for enhancing muscle performance and promoting muscle growth in elderly frail individuals, as well as for improving physical function of this population [75,76]. In addition, resistance exercise can improve body composition by increasing whole body fat oxidation and energy expenditure due to enhanced mitochondrial enzymatic activity and intramyocellular lipid oxidation, which could be particularly helpful for SO patients [77,78]. 

Specifically, a randomized controlled trial was conducted by Stoever et al. (2018) to evaluate the efficacy of a 16-week progressive resistance exercise training program utilizing resistance machines for improving physical function in 28 individuals with SO. The study revealed a significant increase in gait speed of 5% and in short physical performance battery (SPPB) of 13%, as well as in the PPT score of 11%, all of which represent an increase in physical performance. Interestingly, after the 16-week intervention, this population reached the baseline performance values of a non-sarcopenic control group, regarding SPPB and PPT [61]. Similarly, Liao et al. (2018) performed an RCT using 33 women with SO that underwent a 12-week resistance training program utilizing elastic resistance bands, and showed improvements in the physical capacity and function of these patients. More specifically, significant increases were found in the global physical capacity score (GPCS), the gait speed (GS) test, the timed up and go (TUG) test, and the timed chair rise (TCR) task after the 12-week intervention, as well as after a 6-month follow-up period [62]; however, another RCT showed no significant improvement in physical function after 10 weeks of a progressive resistance exercise training program. The program included a high-speed combination of open and closed chain exercises performed using resistance machines and body weight. The study found that both SPPB and GS showed no significant increases or differences compared to the control group [63]. Similarly, De Oliveira Silva et al. (2018) investigated the effects of a 16-week resistance training program utilizing resistance machines in eight SO women and found no influence on the physical function of those patients regarding their performance in TCR and TUG [64]. Balachandran et al. (2014) conducted an RCT in adults with SO, nine of whom participated in a resistance exercise training group and performed strength and hypertrophy exercises for 15 weeks utilizing resistance machines. The group exhibited a 7% non-significant improvement in physical function, as assessed by the modified SPPB, including an improvement in the usual gait speed [65]. Additionally, Villareal et al. (2017) conducted a 26-week clinical trial on 120 obese and frail adults to compare the effectiveness of endurance training, resistance training, and concurrent training, along with a weight-management program. The resistance exercise group, which performed exercises with weightlifting machines, showed a 14% increase in the PPT score, which was lower than that of the concurrent group but equal to that of the resistance exercise group. Moreover, VO2max showed an 8% increase after the 26-week intervention, which was lower than that of the concurrent and the resistance groups [58]. In another clinical trial, the effects of different exercise regimens (endurance, resistance, and concurrent) combined with matched calorie restriction on muscle protein synthesis and myocellular quality were compared in 35 elderly individuals with SO over a 6-month intervention period. The resistance group, which performed exercises with weightlifting machines, showed a 13% increase in the PPT score, which was lower than that of the concurrent group but equal to the endurance group and higher than that of the control group. Additionally, VO2max showed a 7% increase after the 6-month intervention, which was lower than that of the endurance and concurrent groups [59]. Chen et al. (2017) designed an RCT to investigate the influence of different types of exercise (endurance, resistance, and concurrent) on IGF-1 levels and muscle strength performance in 45 elderly adults with SO over an 8-week period. The resistance group, which used weight-training equipment, showed a significantly higher blood IGF-1 concentration after the 8-week intervention compared with the control group. The blood IGF-1 concentration after the 8-week intervention was significantly higher in the resistance training group compared with the control group. Although all the training groups exhibited superior muscle strength compared to the control group, the resistance training group exhibited the greatest difference [60].

### 4.3. Concurrent Training and Its Effects on the Mitochondria in Sarcopenic Obesity

The combined use of resistance and aerobic training, known as concurrent exercise training, has been demonstrated to have superior benefits in improving the functional status of older obese adults compared to either exercise intervention used alone [58]. The addition of resistance exercise following endurance exercise enhances the molecular signaling of mitochondrial biogenesis in human skeletal muscle activated by endurance exercise alone. This mechanism potentially involves cross-talk between the signaling pathways mediated by the mammalian target of rapamycin (mTOR), thus making concurrent training advantageous for enhancing adaptation in muscle oxidative capacity [79]. Additionally, it has been observed that the PGC-1a4, an isoform of peroxisome proliferator-activated receptor-gamma coactivator-1 alpha (PGC-1a) that induces skeletal muscle cell growth, showed a greater increase following a concurrent training program, rather than resistance training alone [80].

In another RCT, women with SO participated in a 24-week combined training program, combining elastic resistance bands exercises with various walking endurance exercises. The results of the study reveal a significant increase in the maximum walking speed (MWS) test, TCR test, and the 2 min step test, representing an overall improvement in the physical function and aerobic endurance of these patients [66]. Similarly, in a study by Kim et al. (2016), the effects of a 3-month concurrent exercise training program with or without nutritional supplementation were examined in 71 women with SO. Resistance and weight-bearing exercises were involved, using resistance elastic bands, a hydraulic exercise machine, and chair exercises, while the aerobic training consisted of endurance training on a stationary bicycle. The results show significant group and time interactions when comparing changes in physical function, walking parameters, and speed before and after the completion of the intervention [67]. In addition, Wohlgemuth et al. (2011) performed a 6-month moderate-intensity concurrent exercise intervention, combining endurance exercise in the form of walking or stationary cycling with resistance exercises using ankle weights, in combination with a weight loss program. Participants in the intervention group experienced an improvement in physical performance as determined by the “time to walk 400 m” test. Additionally, the mitochondrial biogenesis markers PGC-1α and TFAm were increased by the intervention [68]. In the study by Gutiérrez-López et al. (2021), a concurrent training program was implemented in women with SO, which consisted of endurance exercise of moderate intensity, followed by isotonic weightlifting exercises of low intensity that incorporated progressive weight at the end of each section. The results after a 3-month intervention period showed a significant increase in VO2max, as well as fragility parameters and physical fitness in terms of TUG and gait speed. Furthermore, the exercise training program resulted in a pronounced reduction in oxidative damage and an enhancement in antioxidant defense in the SO group [69]. Balachandran et al. (2014) conducted an RCT in adults with SO for 15 weeks. Eight of the volunteers participated in a concurrent training group performing high-speed circuit (HSC) training, an intervention combining circuit training and high-speed resistance exercises utilizing resistance machines. The HSC group exhibited a significant 20% enhancement in physical function, as assessed by the modified SPPB, and an increase in the usual gait speed, while favorable alterations were observed compared to the muscle strengthening group [65]. Villareal et al. (2017) conducted a 26-week clinical trial in 120 obese and frail adults to compare the effectiveness of endurance training, resistance training, and concurrent training, along with a weight-management program. The concurrent group, which performed exercises with weightlifting machines in combination with endurance exercise, showed a 21% increase in the PPT score, which was higher than that of the endurance and the resistance groups. Additionally, VO2max showed a 17% increase after the 26-week intervention period, which was higher than that of the resistance group but lower than that of the endurance group [58]. Another RCT compared the effects of different exercise regimens (endurance, resistance, and concurrent) combined with matched calorie restriction on muscle protein synthesis and myocellular quality in 35 elderly individuals with SO over a 6-month intervention period. The concurrent group, which performed exercises with weightlifting machines in combination with endurance exercise, showed a 20% increase in the PPT score, which was higher than that of the endurance, resistance, and control groups. Additionally, VO2max showed a 16% increase after the 6-month intervention, which was higher than that of the resistance group and equal to the endurance group. Furthermore, the expression of regulatory factors involved in mitochondrial fission, mitophagy, and proteostasis showed a greater reduction in the concurrent group compared to all the other groups [59]. Chen et al. (2017) designed an RCT to investigate the influence of different types of exercise (endurance, resistance, and concurrent) on circulating IGF-1 levels and muscle strength in 45 elderly adults with SO over an 8-week intervention period. The blood IGF-1 concentration was significantly higher in the concurrent training group compared to that of the endurance training and control groups. The concurrent group, which performed moderately intense endurance training in combination with weight-training equipment, showed a significantly higher blood IGF-1 concentration compared to that of the endurance training and control groups. Additionally, the concurrent training group, along with all the other exercise training groups, exhibited superior muscle performance compared to the control group [60].

**Figure 1:**
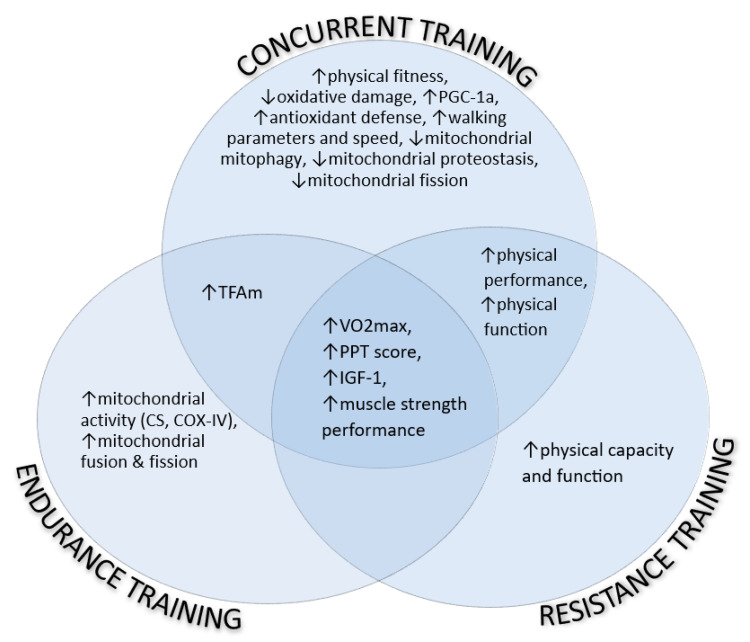
The mitochondrial-related indexes of individuals with SO, influenced by the endurance, resistance, and concurrent training programs independently, as well as their overlaps. COX-IV: cytochrome C oxidase, CS: citrate synthase, IGF-1: insulin-like growth factor-1, PGC-1a: proliferator-activated receptor-gamma coactivator-1 alpha, PPT: physical performance test. ↑: increase, ↓: decrease.

In an aggregated representation of the perceived outcomes, Figure 1 presents the mitochondrial-related indexes of individuals with SO, influenced by the endurance, resistance, and concurrent training programs independently, as well as their overlaps.

### 4.4. “Non-Traditional” Training Protocols and Their Effects on the Mitochondria in Sarcopenic Obesity

Over the last several years, non-traditional training protocols, which might feature a combination of both endurance and resistance training, such as agility training, CrossFit training, flywheel training, whole-body electromyostimulation, and resistive vibration exercise, have become an increasingly popular choice [70,81,82,83]. Research conducted in healthy individuals has shown encouraging effects on VO2max and mitochondrial respiration, as well as physical performance, suggesting a holistic approach to fitness [81,82]; however, the research data regarding the impact of non-traditional training protocols on individuals with SO and other special populations are limited. Kemmler et al. (2016) conducted a training protocol consisting of whole-body electromyostimulation with or without a dietary protein supplement on 50 women with SO aged over 70 years. The training program lasted for 26 weeks and resulted in an increase in gait speed for both training groups; however, the improvement was only significant in the group without the protein supplementation [70].

## 5. Conclusions

Sarcopenic obesity is a multifactorial chronic condition that is characterized by the simultaneous occurrence of skeletal muscle mass loss (sarcopenia) and excess body fat (obesity). It is most commonly detected among the elderly population and is associated with numerous negative health outcomes and comorbidities.

Physical exercise is currently the cornerstone for treating SO and improving mitochondrial function is one of its main targets. Different types of exercise may influence the mitochondrial function in different ways, and this was the focus of the present review. Endurance exercise training was found to be effective in improving mitochondrial biogenesis and enhancing oxidative capacity. This type of exercise stimulates the production of new mitochondria, leading to increased energy production and improved muscle function. Resistance training, on the other hand, is effective in increasing muscle strength and mass, as well as improving mitochondrial function and promoting mitochondrial growth. It has also been shown to increase fat oxidation by enhancing mitochondrial enzymatic activity. Concurrent training, which combines both endurance and resistance exercises, offers a comprehensive approach to address SO. It provides the benefits of both aerobic and muscle strengthening exercises, leading to improvements in muscle mass, strength, and mitochondrial function. This combined exercise training maximizes the potential for muscle growth and metabolic adaptations, ultimately helping to combat SO.

In the studies included in this research, it is generally shown that exercise in the form of endurance, resistance, or concurrent training can be an effective remedy for SO; however, a significant finding emerges, highlighting the efficacy of concurrent training as a more potent strategy for managing SO, based on the positive effects observed on mitochondrial function, in comparison to separate endurance or resistance exercise modalities. This novel insight adds to the existing body of research on the subject, emphasizing the importance of incorporating concurrent exercise as part of integrated exercise programs for addressing SO effectively.

To the best of our knowledge, no other review has examined the role of exercise in relation to mitochondrial function and its impact on SO. This research highlights an underexplored area in the field, emphasizing the necessity for additional clinical trials and studies to further elucidate the intricate mechanisms and potential benefits of exercise interventions in managing sarcopenic obesity through the optimization of mitochondrial function.

This study has potential limitations that need to be acknowledged. Firstly, some of the retrieved studies lacked a control group, restricting the ability to establish a direct comparison between the exercise group and a non-exercise group. Secondly, the limited inclusion of only female participants in some studies could potentially limit the generalizability of the findings to a more diverse population. Understanding potential gender-specific effects could be valuable for a future comprehensive analysis. Lastly, the considerable variation in the training periods across the studies, ranging from two to six months, might have influenced the observed outcomes differently. Despite these limitations, this study contributes to the existing knowledge and serves as a basis for future research.

Overall, SO is closely linked to mitochondrial dysfunction, and endurance training, resistance training, and concurrent exercise training all play an important role in treating this chronic condition by improving mitochondrial function, as these forms of exercise can promote mitochondrial biogenesis, enhance energy production, and preserve muscle mass; thus, utilizing regular exercise programs that include both endurance and resistance training can be an effective strategy for managing SO and improving overall health in these patients.

## Figures and Tables

**Table 1:** The effects of different types of exercise on outcomes related to mitochondrial function in individuals with SO.

Author (Year)	Participants (=n)	Age (Years)	Sex	Type of Exercise	Intervention Period	Main Outcomes
Villareal et al. (2017) [58]	40	70 ± 4	M + F	E.T.	26 weeks	↑VO2max, ↑PPT score
	40	70 ± 5		R.T.		
	40	70 ± 5		C.T.		
Colleluori et al. (2019) [59]	11	71 ± 1	M + F	E.T.	6 months	↑VO2max, ↑PPT score, ↑mitochondrial fusion and fission, ↑mitochondrial activity (CS, COX-IV), ↑TFAm
	12	72 ± 2		R.T.		↑VO2max, ↑PPT score
	12	69 ± 1		C.T.		↑VO2max, ↑PPT score, ↓mitochondrial fission, ↓mitochondrial mitophagy, ↓mitochondrial proteostasis
Chen et al. (2017) [60]	15	69.3 ± 3.0	M + F	E.T.	8 weeks	↑IGF-1, ↑muscle strength performance
	15	68.9 ± 4.4		R.T.		
	15	68.5 ± 2.7		C.T.		
Stoever et al. (2018) [61]	28	M: 71.0 ± 4.27	M + F	R.T.	16 weeks	↑physical performance
		F: 72.2 ± 5.42				
Liao et al. (2018) [62]	33	66.67 ± 4.54	F	R.T.	12 weeks	↑physical capacity and function
Vasconcelos et al. (2016) [63]	14	72 ± 4.6	F	R.T.	10 weeks	(-) physical function
De Oliveira Silva et al. (2018) [64]	8	66.9 ± 3.3	F	R.T.	16 weeks	(-) physical function
Balachandran et al. (2014) [65]	9	71 ± 8.2	M + F	R.T.	15 weeks	↑physical function
	8	71.6 ± 7.8		C.T.		
Park et al. (2017) [66]	25	74.1 ± 6.1	F	C.T.	24 weeks	↑physical function
Kim et al. (2016) [67]	Ex + N: 36	Ex + N: 80.9 ± 4.2	F	C.T.	3 months	↑physical function, ↑walking parameters and speed
	E: 35	Ex: 81.4 ± 4.3				
Wohlgemuth et al. (2011) [68]	6	65.8 ± 6.2	F	C.T.	6 months	↑physical performance, ↑PGC-1a, ↑TFAm
Gutiérrez-López et al. (2021) [69]	30	68.06 ± 5.75	F	C.T.	3 months	↑VO2max, ↓fragility parameters, ↑physical fitness, ↓oxidative damage, ↑antioxidant defense
**“NON-TRADITIONAL” TRAINING PROTOCOL**						
Kemmler et al. (2016) [70]	WB-EMS: 25	WB-EMS: 77.3 ± 4.9	F	WB-EMS	26 weeks	↑gait speed
	WB-EMS&P: 25	WB-EMS&P: 76.4 ± 2.9				

COX-IV: cytochrome c oxidase, CS: citrate synthase, IGF-1: insulin-like growth factor 1, PPT: physical performance test, TFAm: transcription factor A, mitochondria. Ex + N: exercise + nutrition, Ex: exercise only, WB-EMS: whole-body electromyostimulation, WB-EMS&P: whole-body electromyostimulation with dietary supplementation. M: male, F: female. ↑: increase, ↓: decrease, (-): no significant change.

## Data Availability

Not applicable.
